# Clinical and Molecular-Genetic Insights into the Role of Oxidative Stress in Diabetic Retinopathy: Antioxidant Strategies and Future Avenues

**DOI:** 10.3390/antiox9111101

**Published:** 2020-11-09

**Authors:** Silvia M. Sanz-González, José J. García-Medina, Vicente Zanón-Moreno, María I. López-Gálvez, David Galarreta-Mira, Lilianne Duarte, Mar Valero-Velló, Ana I. Ramírez, J. Fernando Arévalo, María D. Pinazo-Durán, on behalf of the Valencia Study Group on Diabetic Retinopathy (VSDR) Report number 4

**Affiliations:** 1 Ophthalmic Research Unit “Santiago Grisolía”, Fundación Investigación Sanitaria y Biomédica (FISABIO), Ave. Gaspar Aguilar 90, 46017 Valencia, Spain; sanz.gonzalez.sm@gmail.com (S.M.S.-G.); jj.garciamedina@um.es (J.J.G.-M.); vczanon@universidadviu.com (V.Z.-M.); vavema@alumni.uv.es (M.V.-V.); dolores.pinazo@uv.es (M.D.P.-D.); 2 Cellular and Molecular Ophthalmo-Biology Group, University of Valencia, Ave. Blasco Ibañez 15, 46010 Valencia, Spain; 3 Spanish Net of Ophthalmic Research “OFTARED” RD16/0008/0022, of the Institute of Health Carlos III, 28029 Madrid, Spain; maribel@ioba.med.uva.es (M.I.L.-G.); dgalarreta@saludcastillayleon.es (D.G.-M.); 4 Department of Ophthalmology, General University Hospital Morales Meseguer, Ave. Marques de los Velez, s/n 30008 Murcia, Spain; 5 Department of Ophthalmology and Optometry, University of Murcia, Edificio LAIB Planta 5ª, Carretera Buenavista s/n, 30120 El Palmar Murcia, Spain; 6 Area of Health, Valencian International University, Calle Pintor Sorolla 21, 46002 Valencia, Spain; 7 Department of Ophthalmology, The University Clinic Hospital, Ave. Ramón y Cajal 3, 47003 Valladolid, Spain; 8 Department of Ophthalmology, Complexo Hospitalar “Entre Douro e Vouga”, 4520-211 Santa Maria da Feira, Portugal; lilianne.duarte@chedv.min-saude.pt; 9 Department of Immunology, Ophthalmology and Otorrinolaringology, Faculty of Optics and Optometry, Universidad Complutense, Calle Arcos de Jalón 118, 28037 Madrid, Spain; 10 Instituto de Investigaciones Oftalmológicas “Ramón Castroviejo”, Faculty of Medicine, Universidad Complutense, Plaza Ramón y Cajal, s/n 28040 Madrid, Spain; 11 Wilmer s Eye Institute at the Johns Hopkins Hospital, Baltimore, MD 21287, USA; arevalojf@jhmi.edu

**Keywords:** type 2 diabetes mellitus, retinopathy, oxidative stress, antioxidants, omega-3 fatty acids, candidate biomarkers, prevention of blindness

## Abstract

Reactive oxygen species (ROS) overproduction and ROS-signaling pathways activation attack the eyes. We evaluated the oxidative stress (OS) and the effects of a daily, core nutritional supplement regimen containing antioxidants and omega 3 fatty acids (A/ω3) in type 2 diabetics (T2DM). A case-control study was carried out in 480 participants [287 T2DM patients with (+)/without (−) diabetic retinopathy (DR) and 193 healthy controls (CG)], randomly assigned to a daily pill of A/ω3. Periodic evaluation through 38 months allowed to outline patient characteristics, DR features, and classic/OS blood parameters. Statistics were performed by the SPSS 24.0 program. Diabetics displayed significantly higher circulating pro-oxidants (*p* = 0.001) and lower antioxidants (*p* = 0.0001) than the controls. Significantly higher plasma malondialdehyde/thiobarbituric acid reactive substances (MDA/TBARS; *p* = 0.006) and lower plasma total antioxidant capacity (TAC; *p* = 0.042) and vitamin C (0.020) was found in T2DM + DR versus T2DM-DR. The differential expression profile of solute carrier family 23 member 2 (SLC23A2) gene was seen in diabetics versus the CG (*p* = 0.001), and in T2DM + DR versus T2DM − DR (*p* < 0.05). The A/ω3 regime significantly reduced the pro-oxidants (*p* < 0.05) and augmented the antioxidants (*p* < 0.05). This follow-up study supports that a regular A/ω3 supplementation reduces the oxidative load and may serve as a dietary prophylaxis/adjunctive intervention for patients at risk of diabetic blindness.

## 1. Introduction

Diabetes mellitus (DM) is a multifactorial-polygenic disease characterized by chronic hyperglycemia and altered metabolism of carbohydrates, lipids, and proteins. There are two major clinical forms: Insulin-dependent DM (type 1: T1DM) and non-insulin-dependent DM (type 2: T2DM), being the latter the most common presentation, accounting for 90% of all diabetics [1]. DM displays alarming growth rates worldwide, being a serious health concern with an estimated number of affected people of 600 million by the year 2030 [2,3]. 

Microvascular changes are clinically manifested during the natural course of DM. Population studies reported that 20% of diabetics had retinopathy (DR) at the time of diagnosis [4]. The main pathogenic mechanisms in the diabetic retina include the activation of oxidative stress (OS), advanced glycation end-product generation, inflammation, activation of protein kinase C, polyol, and hexosamine pathways, etc. [5]. In addition, a series of endogenous/exogenous risk factors have been pointed as contributing highly to the DR initiation and progression [6]. 

The incomplete reduction of molecular oxygen (O_2_) leads to the formation of reactive oxygen species (ROS) with unpaired electrons in its outermost orbital, making these molecules extremely unstable and reactive. The generation of superoxide anion (O_2_^−^) and hydrogen peroxide (H_2_O_2_) is prone to start the free radical chain reaction due to hydroxyl radical formation (OH^−^), by the Fenton and Haber Weiss chemical reactions [7,8]. Furthermore, nitric oxide (NO) is a non-reactive free radical implicated in a variety of biological functions, such as vasodilation, immune response, neurotransmission, and apoptosis [9,10].

It is well known that redox status influences the regulation of intracellular signaling with enormous repercussions in health and disease [11]. In this context, epidemiologic and experimental studies demonstrated that DR is noticeably related to OS [5,12,13,14,15]. 

The concern of nutritional supplements in ocular health and disease has been largely addressed [16,17]. Nutritional control has been proposed as a potential intervention for slowing DR initiation/progression, but studies on dietary assessments in diabetics showed contradictory results [18,19,20,21,22,23]. Moreover, nutraceuticals cannot replace eating adequate amounts of a high variety of foods, but more than half of Americans and many Europeans take at least one dietary supplement on a regular basis [16,17]. These may be especially beneficial to individuals with poor nutrition caused by a variety of processes, including DM. International science-based regulatory considerations in nutrition and nutraceutical supplements are needed to update its recommendations for the ophthalmological population [18,19,20,21,22,23]. Antioxidant (A) vitamins (vit) are essential for a wide variety of biological functions. Among them, the reduced form of ascorbic acid (AscH2; L-ascorbic acid; vit C), a hydrophilic vitamin, enzymatic cofactor, and ROS scavenger, is involved in the protection against a variety of disorders. Abnormal dietary intake and/or plasma levels of vit C (deficiency defined as a concentration equal of less than 35 µmol/L) have been associated to a higher risk of getting sick, including the development of xerosis and other ophthalmic pathologies [24]. In this concern, vit C absorption and distribution to tissues and organs is achieved by active transport across the cell membranes. Two specific protein transporters encoded by the sodium-dependent vit C transporter genes, SLC23A1 and SLC23A2 [25], are involved in this fact. The latter is responsible for the regulation of the intracellular levels of vit C to protect cells from OS, as well as to promote the type I collagen maturation. In fact, genetic variations in the SLC23A2 gene expression modify the vit C availability [24,25].

Given that early diagnosis can delay or prevent DR-related blindness, there is considerable interest in exploring the role of its molecular diagnostics [17,18]. Up to today, studies on DR biomarkers have dealt with pathogenic processes and risk factors involved in DR development/progression [2,3,5,26,27]. The need for more accurate diagnostics that allow advance ophthalmologic and vision care is currently providing a critical window of opportunity for diabetics. The present study deals with improving knowledge about the classical and new potential diagnostic/prognostic candidate biomarkers to verify/validate them by using various endpoints like ophthalmic indicators, classical blood biochemistry, oxidative and antioxidant parameters, as well as the *SLC23A2* gene. The secondary endpoint was to analyze the effect of a course of A and omega 3 fatty acids (A/ω3) in the form of mono-combination on the OS presumptive biomarkers, in order to better managing the diabetic eyes. 

## 2. Materials and Methods 

### 2.1. Community-Based Study Design

The study adheres to the Declaration of Helsinki (Edinburgh, 2000), the Ethics Committee standards of the study centers (no. 12/33), and the Spanish Agency of Medicines and Medical Devices (AEMPS). All requirements for clinical research and to maintain the privacy of the data obtained were met. A prospective interventional open-label multicenter case-control study was conducted between 2014–2015/2018–2019. Part of the study design and sampling methodology has been described in detail [19]. The main purpose was outlining T2DM patient characteristics, risk factors, precise blood-based biochemical parameters, and typical DR features to identify novel clinical and molecular-genetic biomarkers for DR. The second objective was to look at the effects of a course of (A/ω3) in the health and vision care among T2DM patients with (+) and without (−) DR. 

### 2.2. Participants

575 potential participants of both sexes aged 25–80 years [300 T2DM patients and 275 healthy controls (CG)] were carefully recruited by general practitioners and ophthalmologists (listed at the end of the manuscript) to ensure consistency and efficacy of the study sample, interviewed according to the study inclusion/exclusion criteria (Table 1) and appointed to the first selection step.

### 2.3. Screening Procedures 

#### 2.3.1. Selection and Appointment Schedules

Baseline data were recorded as: Socio-demographics, personal/familial background (DM duration and treatment) and personal characteristics [nutrition-mediterranean diet (MedDiet) adherence by the validated 14-items questionnaire; Prevención con Dieta Mediterránea (PREDIMED), as previously reported [19], body mass index (BMI) and regular physical activity classified as to practice (1) or no (2) exercise. At the first visit, patients underwent an ophthalmological examination in both eyes: best-corrected visual acuity (BCVA), intraocular pressure (IOP), ocular fundus examination/retinography, and spectral domain optical coherence tomography (SD-OCT).

**Figure 1:**
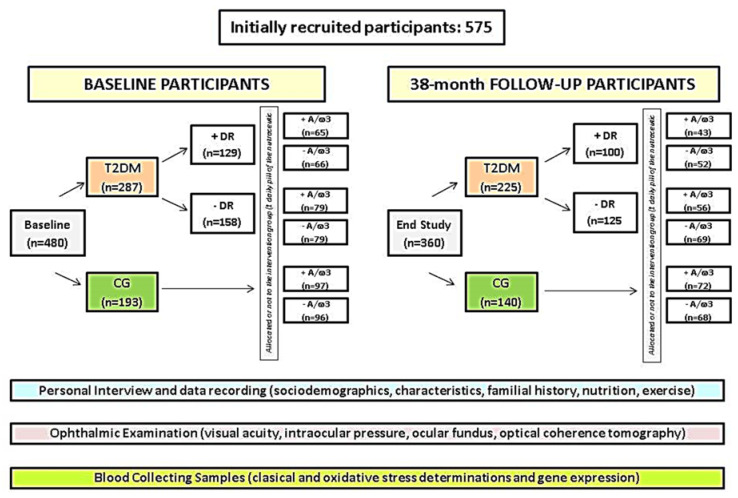
Flowchart of the recruitment, classification, and random assignation of the nutraceutical regimen in the study participants.

Of the initial recruitment, 480 suitable participants were verbally informed and signed on the consent form. The participants were then classified into: T2DM patients (*n* = 287) and CG (*n* = 193) (Figure 1). Diabetics were also sub-classified according to having retinopathy “+DR” (*n* = 129) or not “−DR” (*n* = 158). (Figure 1).

**Figure 2:**
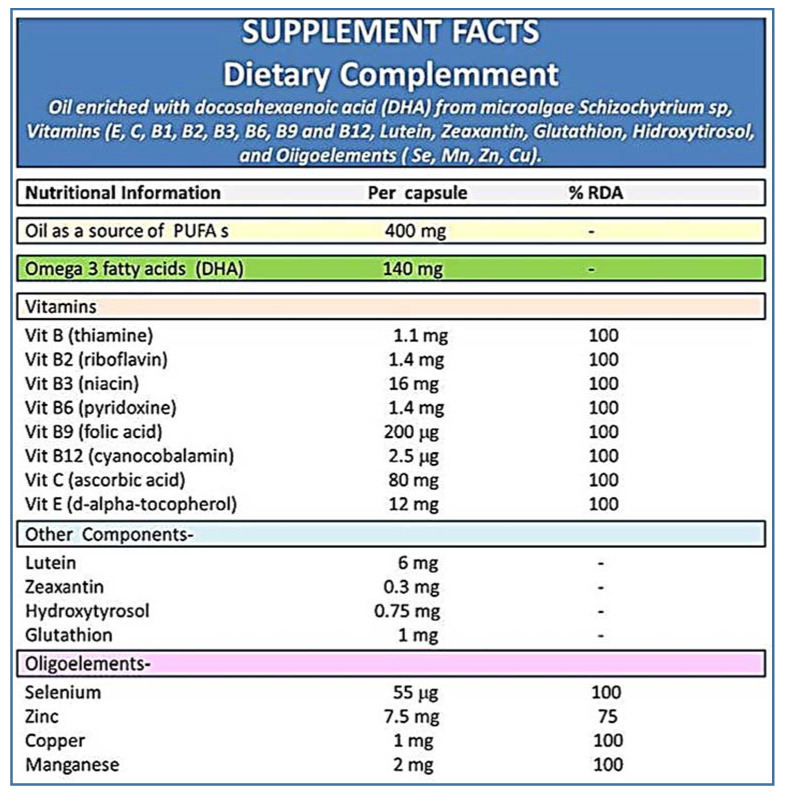
Oral supplement formula.

Diabetics and controls were homogeneously divided into those randomly assigned to “a pill of A/ω3 per day: +A/ω3” or “no-pill condition: −A/ω3”, as reflected in the Figure 1. Pills containing vit (E, C, B1, B2, B3, B6, B9, B12), lutein, zeaxanthin, glutathione (GSH), hydroxytyrosol, trace elements (Se, Mn, Zn, Cu) and docosahexaenoic acid (DHA), commercialized as Nutrof Omega^®^ in packs of 35 capsules of 33.49 g each (Laboratorios Thea SA, Barcelona, Spain) were gently provided by the manufacturer and given to the participants without any charge (Figure 2). Ophthalmic visits were scheduled every 6 months throughout the 38-months follow-up. Patients with proliferative DR (PDR) or diabetic macular edema (DME) were excluded by its special vulnerability to severe complications and the need to receive specific treatments immediately. 

#### 2.3.2. Ophthalmologic Procedures

Ocular examination in each eye included: BCVA, ocular fundus inspection/photographs (ImageNet; Topcon, Barcelona, Spain), and SD-OCT (Carl Zeiss Meditec, Madrid, Spain). OCT parameters were measured by the direct cross-sectional imaging device. Retinographies and SD-OCT data were evaluated by retina specialists to obtain optimal nonproliferative DR (NPDR) diagnosis-grading in each eye, according to the Early Treatment of Diabetic Retinopathy Study (ETDRS) [28] severity scale. The presence and number of microaneurysms/hemorrhages, venous beading, and intraretinal microvascular abnormalities were considered for DR diagnosis/progression. Worsening was considered an augmentation of the above signs. As reflected in Table 1, those patients with new vessel growth on the retinal surface (PDR) and the presence of focal-diffuse DME were also excluded from final data processing. 

#### 2.3.3. Biosample Processing

Blood sampling was scheduled from baseline and every 6 months (during the 38-month follow-up) to biochemical and molecular-genetic probes. Antecubital vein blood was collected into 4.5 mL ethylene-diamine-tetra-acetic acid (EDTA) or sodium citrate vacutainer tubes (Becton Dickinson, Auckland, New Zealand), as an anticoagulant, under fasting conditions at 8:00 a.m. One EDTA tube (purple cap) was used to determine glucose, glycosylated hemoglobin (HbA1c), and plasma vit C concentrations. The other EDTA tube was used to analyze gene expression. Finally, the sodium citrate tube (purple cap) was centrifuged at 3000 rpm/10 min to obtain the plasma fraction, which was aliquoted and stored at −80 °C until processing. This plasma was used to analyze malondialdehyde (MDA), total antioxidant capacity (TAC), and total glutathione (GSH). All experiments were performed in duplicate. The determinations of glucose and HbA1c were carried out in the Department of Clinical Analysis of the University Hospital Dr. Peset (Valencia, Spain). To complete the analytical proceedings, the remaining experiments were performed in the laboratories of the Ophthalmic Research Unit “Santiago Grisolía” (Valencia, Spain).

-Determination of lipid peroxidation by-products. MDA/thiobarbituric acid reactive substances (TBARS): MDA/TBARS. It was assayed at high temperature (90–100 °C) under acidic conditions and extracted with butanol. Fluorescence was measured in duplicate at 544 nm excitation, 590 nm emission in relation to standard samples fluorescence. The concentration was calculated by extrapolating all data in the standard curve, as reported [19,29].
-Determination of TAC. This was measured by the antioxidant assay kit (Ref: 709001, Cayman Chemical Company, Ann Arbor, MI, USA) based on the antioxidant capacity to inhibit the 2,2′-azino-di-[3-ethylbenzthiazoline sulphonate] oxidation to 2,2′-azino-di-[3-ethylbenzthiazoline sulphonate] radical solution by the metmyoglobin, as published [19,30].
-Determination of total GSH. A modification was done [19] of the method, firstly reported by Tietze [31]. The OxiSelectTM Total GSH (GSSG/GSH) kit was utilized (Cell Biolabs, INC, Ref: STA-312. Madrid, Spain). Global thiol reagent, 5-5′-dithiobis [2-nitrobenzoic acid] (DTNB) reacts with GSH to form both the 412 nm chromophore, 5-thionitrobenzoic acid (TNB), and the disulfide product (GS-TNB). The GS-TNB was reduced through an enzymatic reaction catalyzed by the GSH reductase and β-nicotinamide adenine dinucleotide phosphate (NADPH). Thus, a second TNB molecule was released by recycling the GSH. Any oxidized GSH (GSSG) initially presented in the reaction mixture or formed from the mixed disulfide reaction of GSH with GS-TNB is reduced to GSH, and measured, as described [19,31].
-Determination of the glycemic profile [fasting glucose and HbA1c were performed by 2 different automated chemistry analyzers in the Department of Clinical Analysis of the main study center, as follows: (1) Abbott kits manufactured for use with the Architect c8000 (Abbott Laboratories; Abbott Park, IL, USA) and (2) Arkray AU 4050 (Arkray Global Bunisess Inc., Kyoto, Japan), respectively.
-Determination of plasma vitamin C (vit C). Fresh frozen plasma aliquots were thawed and acidified by adding perchloric acid and diethylenetriaminepentaacetic acid (DTPA; a strong trace metal chelator) for avoiding the ascorbate to destabilize. After centrifugation, supernatants were treated with Tris (2-carboxyethyl) phosphine (TCEP; a potent thiol-free reducing agent) to gather any rest of the ascorbate that became oxidized during previous proceedings. The concentration of vit C was determined by high-performance liquid chromatography (HPLC) with electrochemical detection, as previously reported [32,33]. Briefly, a Shimadzu HPLC System (Shimadzu Scientific Instruments, Columbia, MD, USA) that was equipped with a 5 µM YMCPack ODS-AQ column (Waters Corp., Milford, MA, USA) and a Coulochem III electrochemical detector (ESA, Chelmsford, MA, USA), under reversed-phase conditions was used. Sampling injection volume was 5 µL, and compounds were eluted over an 18 min runtime at a flow rate of 0.6 mL/min, following the method described by Li [32] with minor modifications [33]. Plasma vit C concentrations were expressed as mean (SD) in µmol/mL, taking into consideration the Linus Pauling Institute recommendations (https://lpi.oregonstate.edu/mic/vitamins/vitamin-C) of consuming sufficient vit C to obtain at least a circulating concentration of 60 μmol/L, as well as international guidelines as follows: <11 μmol/L indicate severe deficiency; 23–50 μmol/L inadequate; 50–70 μmol/L adequate; and >70 μmol/L is deemed saturating.
-Gene expression assays. Whole blood samples were obtained from each participant and collected into EDTA tubes. Total RNA was isolated from blood samples by the Trizol method. Then, 300 ng of total RNA (integrity number—RIN > 7) were converted into cDNA by reverse transcription using the High-Capacity RNA-to-cDNA™ Kit (Applied Biosystems, Foster City, CA, USA). The relative SLC23A2 gene expression was analyzed by real-time PCR, using a 7900HT Sequence Detection System (SDS; Applied Biosystems^®^, Madrid, Spain). TaqMan gene expression assays were used for both target (SLC23A2) and internal control (18S rRNA) genes (Applied Biosystems^®^, Spain). Samples were assayed in duplicate. The expression values were calculated by the double delta Ct formula, as previously reported [34,35], and the results were expressed as fold changes in gene expression for each group and subgroup, at baseline.

### 2.4. Statistical Analysis

The “Microsoft Excel” program and “IBM Statistical Package for the Social Sciences” SPSS V.24.0 program (IBM SPSS Statistics for Windows, Version 24.0. Armonk, NY, USA) were used. For continuous variables, non-parametric (Mann–Whitney U) and parametric (*t*-test) statistics were utilized, and the results were expressed as mean ± standard deviation (SD). Categorical variables were expressed as percentages. A *p*-value of less than 0.05 was considered statistically significant, which was adjusted by a Bonferroni correction when pairwise comparison in multiple groups was conducted. The non-parametric Spearman’s Rho correlation test (or correlation coefficient) was used to evaluate the power of association between 2 specific variables (the value *r* = 1 means a positive correlation, and the value *r* = −1 means a negative correlation).

## 3. Results

Out of the suitable initial 480 participants, 365 (rate of response from baseline: 76%) completed their 38-month study course (Figure 1). The overall dropout rate was 24%. Deterioration of health/lack of motivation (7%), confusing ocular fundus or retinographies, and/or progression to PDR or DME (10%), and blood samples failing to show any data (7%) constituted common causes of withdrawn.

Table 2 shows the main socio-demographic data, medical history, and laboratory parameters of the study participants. 26% of the baseline population had DR. Lead time between the DM diagnosis and the last study visit was 22 (5) years in the +DR and 15 (3) years in the −DR (*p* = 0.002).

Eating habits were addressed during the study course to check whether the participants had nutrient adequacy and adherence to the MedDiet. Higher nutritional control was observed in the T2DM − DR (41%) than in the T2DM + DR (30%) (*p* = 0.001). Moreover, a higher percentage of diabetics +DR (32%) displayed a lower MedDiet questionnaire score as compared to the T2DM − DR patients (19%) (*p* = 0.0001). Significantly higher BMI was seen in diabetics vs. the CG (*p* = 0.001).

No significant differences were observed between the T2DM patients and the CG, regarding age, gender, and physical exercise routine, but statistically significant differences (*p* = 0.000001) in glucose and HbA1c determinations were detected between the main study groups (Table 2).

**Figure 3:**
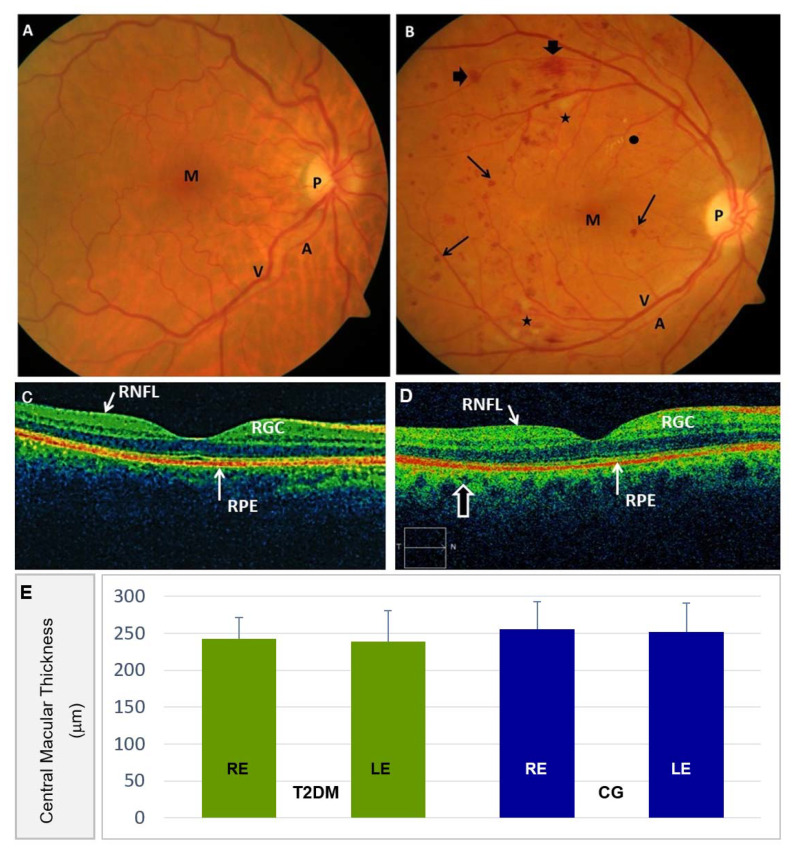
Ocular Fundus imaging. (**A**) Standard color fundus retinography showing 30° of the posterior eye pole (including the optic nerve head and the macula) of the RE from a participant of the CG (P: Optic nerve papilla; M: Macula; A: Retinal artery; V: Retinal vein). (**B**) Standard color fundus retinography showing 30° of the posterior eye pole of the RE displaying the clinical microvascular angiopathy manifestations of a T2DM patient with nonproliferative diabetic retinopathy (NPDR). Note the microaneurysms and abundant microhemorrhages (arrowthin), as well as macrohemorrhages (arrowhead). Scarce hard exudates (●) and cotton-wool spots (*) (being the latter grey-white discolored plaques located in the retinal nerve fiber layer, corresponding to local ischemic areas) were detected. The same abbreviations reflected in A for the main retinal structures can be detected in this pathologic image. No macular alterations were detected in this NPDR retinography. (**C**) Spectral Domain Optical Coherence tomography (SD-OCT) of a CG participant. Normal macular structural details of the same healthy participant as referred to in A. Among other retinal structures, the retinal nerve fiber layer (RNFL), retinal ganglion cell (RGC), and the retinal pigment epithelium (RPE)/Bruch’s membrane complex can be identified. (**D**) The microaneurysms and the hemorrhages (identified as hypo-reflective lesions) were present in the SD-OCT scans of the above diabetic patient, as referred in B. No macular changes and no neovascularization were detected in the NPDR images. (**E**) Central Macular Thickness in the right eye (RE) and left eye (LE) of the T2DM participants, as compared to the CG.

Ocular fundus was explored and photographed in the CG participants, showing a normal appearance without any DR signs (Figure 3A), in contrast to the diabetics with mild-to-moderate retinal complications, as reflected in the ocular fundus of Figure 3B.

Furthermore, the series of ocular fundus examinations of the T2DM group revealed a mild increment in NPDR development and progression with respect to baseline. Among the participants who completed the study altogether, those with retinopathy showed impairment of retinal affectation grading (54%: Mild DR; 35%: Moderate DR; 11%: Severe DR) from baseline (76% mild; 21% moderate; 3% severe).

Morphologic and morphometric evaluation of the OCT images was performed on the T2DM patients and the CG individuals (Figure 3C,D). Representative OCT images, as shown in Figure 3C,D, did not show noticeable differences in central retinal thickness from each eye when the diabetic patients and the control individuals were compared (Figure 3E). We have to emphasize that this tool improved our capacity to document diabetic retinal lesions in the posterior eye pole. Table 3 shows the main parameters of the ophthalmologic examination in the study participants. These data revealed noticeable differences in structural/functional values between groups, distributed into participants assigned to the A/ω3 regimen versus the no supplemented ones.

A relevant point of this follow-up study was the evaluation of DR progression. In fact, the ETDRS grading scale [28] (that is more popular among the researchers that other similar DR classification systems) was used in the present work to define DR progression. The presence of hemorrhages, hard exudates, and cotton-wool spots was equivalent to the ETDRS level 35 of a mild NPDR [28]. Moderate and severe NPDR forms have also been conveniently staged, as reflected in Figure 3.

From the 129 T2DM patients with DR (NPDR forms) at baseline, 22% showed a cumulative incidence of DR impairment at the end of follow-up, the majority of them (13%) corresponding to mild NPDR stages, 5% to moderate stage, and 4% corresponding to the severe NPDR form. Among the diabetics that showed clinical signs and symptoms of retinopathy progression, 65% pertained to the −A/ω3 subgroup. From the 158 initial T2DM patients without NPDR, (27%) developed DR at the end of the study, 12% mild; 9% moderate; 5% severe. Among the diabetics that initially did not suffer DR, 58% pertained to the −A/ω3 subgroup as shown in the Table 4.

The OS markers were assayed in plasma samples from the groups and subgroups of participants. At baseline, comparison of the CG and the T2DM patients reflected significantly higher plasma levels of MDA/TBARS [2.9 (0.1) µM vs. 2.1 (0.2) µM (*p* = 0.001)] and significantly lower plasma levels of TAC [2.3 (0.1) mM vs. 3.1 (0.3) mM (*p* = 0.001)], GSH [2 (0.2) µM vs. 2.8 (0.2) µM (*p* = 0.002)] and vit C [40 (19) µM/mL vs. 56 (20) µM/mL (*p* = 0.001)]. Therefore, diabetics displayed significantly higher circulating pro-oxidant markers and lower antioxidant levels than the non-diabetic study population. Furthermore, significantly higher plasma levels of MDA/TBARS, lower TAC, as well as decreased vit C were observed in the T2DM + DR with respect to the T2DM − DR patients, both at baseline and at the end of follow-up. Table 5 illustrates the effects of the A/ω3 administration in mono-combination on biochemical blood traits, as the patients were categorized in relation to their response to the nutraceutical regimen. Therefore, participants randomly assigned to the oral +A/ω3 improved their OS background with statistical differences between the T2DM + DR vs. T2DM − DR patients throughout the 38-month follow-up (Table 5). A regular A/ω3 supplementation course reduced the OS by conferring a potential neuroprotective effect to the diabetic retina, which is a priority and challenging topic for our current research and the foreseeable future.

In our study population, we observed that aging, higher DM length, bad metabolic control (as reflected by the elevated levels of glycemia and HbA1c) in the presence of significant oxidative load were frequent findings that helped to identify diabetics at risk of DR and DR progression. We also found that overweight patients had poorer overall glycemic control also suffered a more advanced stage of NPDR than the normal-weight diabetics throughout the 38-month follow-up.

**Figure 4:**
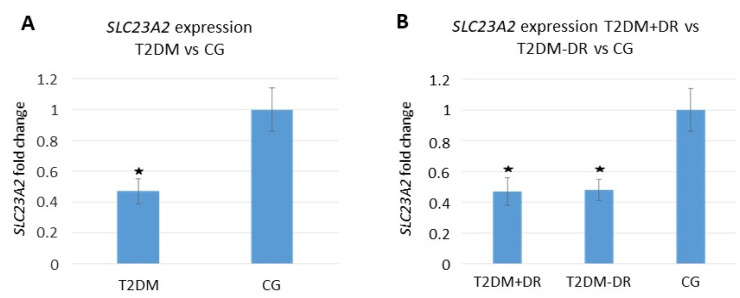
Plasmatic SLC23A2 relative expression at baseline. (**A**) T2DM vs. CG; (**B**) T2DM + DR vs. T2DM − DR vs. CG. (SLC23A2: Solute Carrier Family 23 Member 2 gene; T2DM: Type 2 diabetes mellitus, +/−DR: With/without diabetic retinopathy, CG: Control group. *: *p* < 0.05 statistically significant).

Regarding the gene expression assays, we detected a differential expression profile of the SLC23A2 gene (responsible for the regulation of the intracellular levels of vit C) between the main groups and subgroups of the study participants at baseline (Figure 4).

When corrected, these data for multiple testing, significantly lowered SLC23A2 gene expression (fold change) in the T2DM vs. the CG (*p* < 0.05) and in the T2DM +DR vs. −DR (*p* < 0.05) was detected. Furthermore, we found that the SLC23A2 gene was down-regulated in T2DM patients, and it was associated with significantly lower vit C plasma levels (*p* = 0.001). The above association remained statistically significant (*p* < 0.05) even after a multivariate adjustment for major potential confounders (sex, age, eating habits, and BMI) was carried out in the data processing.

In addition, we analyzed the correlation between the main OS markers and the SLC23A2 gene expression in blood samples of the study participants at baseline. Spearman Rho correlation between the plasma MDA/TBARS values and SLC23A2 blood gene expression was statistically significant [0.290 (*p* = 0.026) for the T2DM and 0.188 (*p* = 0.048) for the CG, and 0.651 (*p* = 0.030) for the T2DM + DR vs. 0.711 (*p* = 0.0002) for the T2DM-DR]. Spearman Rho correlation between the plasma TAC and SLC23A2 blood gene expression was statistically significant for the comparison of diabetics and controls [0.294 (*p* = 0.027) in the T2DM vs. 0.038 (*p* = 0.012) in the CG], as well as for the T2DM + DR patients [0.0427 (*p* = 0.001)], but no significant correlation was detected for the T2DM-DR patients [0.084 (*p* = 0.144)].

The final rate of compliance with the A/ω3 regimen was 89% for the T2DM and 80% for the CG. The highest response was for the T2DM + DR group than those from the T2DM − DR patients. There were no observed/reported side effects of the oral supplementation, except a few cases of mild gastrointestinal complaints.

## 4. Discussion

The main results from this population study from baseline to the 38-month follow-up were: (1) DR progression was more likely with higher DM duration, BMI, and Hb1Ac in the context of outstanding OS, and (2) Noticeable amelioration over six consecutive screening intervals, was detected in major hematological parameters and OS markers in the T2DM + DR patients randomly assigned to the oral A/ω3 regimen, as compared to the non-supplemented participants.

Several authors reported that metabolic and risk factors control ameliorated DR prognosis [1,2,3,4,5,6,19,28]. In the current study, we planned to analyze classic risk factors (T2DM duration, sedentary lifestyle, eating habits, and BMI), as well as blood parameters (glucose/HbA1c) and findings from the circulating candidate DR predictors (MDA/TBARS, TAC, GSH, vit C, and the SLC23A2 gene). Recent reports established that DR prevalence in Spain in the T2DM patients depends on the study population, ranging from 15% [27] to 26% [28,36]. From our initial 480 participants, 26% had DR. In addition, the mean T2DM lastingness at 38-months follow-up was 15 (3) years in −DR patients vs. 22 (5) years for the +DR patients. Nevertheless, additional factors also play critical roles in managing DR at any stage [1,2,3,4,5,6,19,26,27,28], with a special interest in diabetics without clinically detectable retinopathy and those suffering from the early DR stage [37,38].

Nutrition is a pivotal component for diabetic patients care. Regarding DR, researchers have described the role of micronutrients (including A/ω3), macronutrients, several food groups, and dietary aspects and lifestyle, such as the MedDiet, in the initiation and progression of DR [4,5,6,18,19,20,21,22,23,24]. Despite this, findings remain inconclusive, and current evidence does not inform about the specific dietary components that are likely to reduce (or increase) the risk of DR. Moreover, new pharmacologic therapies for DR are urgently needed [4,19,26,27,28].

A growing paradigm in managing DR is the relevance of endogenous pathogenic mechanisms, such as OS and its downstream effectors [4,12,13,14,15,19]. A variety of plasmatic OS biomarkers (MDA/TBARS, TAC, vit C, vit E, and ω3) have been reported in diabetics [7,12,13,14,19,26,27,39,40]. Furthermore, Pan et al. interestingly reported a combinatorial molecular signature that may potentially be used as a marker of PDR [41]. Regarding the latter report, we similarly have described a differential profile of circulating OS markers in the T2DM patient’s with respect to the non-diabetic individuals, but our diabetic population was only constituted by NPDR patients.

There is growing interest in identifying genes that could trigger OS in diabetics. Our experiments allowed us to demonstrate a statistically significant differential expression profile of the SLC23A2 gene between the T2DM patients and the non-diabetic participants, as well as between the T2DM + DR vs. −DR patients. Being this gene the regulator of vit C intracellular levels (for protecting the cells from OS) and the promoter of type I collagen maturation [24,25], we also found that the SLC23A2 gene was down-regulated in T2DM patients and highly associated with significantly lower vit C plasma levels. Multivariate adjustment for major potential confounders (sex, age, eating habits, and BMI) was carried out in the data processing, and the association of lower SLC23A2 expression levels with lower vit C plasma concentration remained statistically significant in our study population (*p* < 0.05). Shaghaghi et al. [42] emphasized the necessity to consider the genetic variation of vitamin C transporters in clinical and epidemiologic studies regarding complex diseases. Encouraged by our results, we strongly suggest the connecting role of the SLC23A2 gene down-regulation and the inappropriate vit C bioavailability, with redox imbalance in the context of chronic hyperglycemia and DR. In this concern, Murgia and Adanski [43] also reported that genetic variation alters nutritional requirements, and that nutritional genomics provides an outstanding challenge for biomedical research, as in DR.

In addition, a significant plasmatic decrease in MDA/TBARS, as well as an increase in TAC, was found in the T2DM +A/ω3 and the CG +A/ω3 during the study period. Inclusion of multiple components in the tested formula [vit (E, C, B1, B2, B3, B6, B9, and B12), lutein, zeaxanthin, GSH, hydroxytyrosol, trace elements (Se, Mn, Zn, and Cu) and DHA] theoretically targeting multiple and overlapping pathogenic mechanisms implicated in DR, strongly support a wider counter-response [7,12,13,14,19,44,45,46]. In fact, the European Union ROS consortium (EU-ROS) stated in 2017 that many diseases had been found to be associated with OS, and subsequently, the theory that OS-related pathologies can be corrected by antioxidant therapy was launched [47]. However, while experimental studies support this theory, clinical studies still generate controversial results [48,49]. In our opinion, the possibility remains that biotherapies based on the redox changes occurring in diabetics may help to protect the retina. In the latter concern, an interesting spectrum of presumptive factors for DR protection has been ultimately proposed, including outstanding antioxidants [50,51,52,53].

From a clinical viewpoint, retinal microvascular changes found in the NPDR eyes are related to basement membranes thickening, pericyte loss, capillary occlusion, and microaneurysms, that can progress towards ischemia, macular edema, and neovascularization [4,5,19,36,37,38]. In this context, image analysis of retinal lesions can provide qualitative and quantitative data to accurately diagnosing and grading DR. Our data suggest relatively lower rates of progression of DR in our cohort during the 38-month follow-up. Regarding the participants that completed the study, only 11% of the T2DM patients +DR progressed to severe NPDR forms. Among the participants assigned to the A/ω3 supplementation, the impairment was noticeably less than in the non-supplemented participants. From the 129 T2DM patients with DR (NPDR forms) at baseline, 22% showed a cumulative incidence of DR impairment at the end of follow-up, the majority (18%) corresponded to the mild-to-moderate NPDR stages. Among the diabetics that showed clinical signs and symptoms of retinopathy progression, 65% pertained to the −A/ω3 subgroup. Moreover, from the 158 initial T2DM patients without NPDR, 27% developed DR at the end of the study. In addition, the majority of these pertain to mild-to-moderate stages of the disease (21%), but 5% progressed to the severe NDPR. Among the T2DM that initially did not suffer DR, 58% were concerned with the −A/ω3 subgroup.

Data also showed that a reduction of hyperglycemia, HbA1c, and BMI may have a beneficial effect on the progression to more severe DR forms. We also confirmed that a regular A/ω3 supplementation course reduces the oxidative load, confers beneficial effects as opposed to detrimental ones, and may potentially act as a dietary prophylaxis/adjunctive intervention for high-risk diabetics. The OCT imaging technique provides retinal cross-section images within the micrometer resolution, allowing the evaluation of structural characteristics of the healthy and pathologic retina [54]. It is widely known that the OCT examination qualitatively and quantitatively improves the preclinical diagnosis of retinal changes occurring in diabetics [13,19,55,56,57,58,59,60]. It was reported in T2DM -DR patients that the foveal and temporal retinal thickness appeared significantly reduced and that a higher HbA1c level was associated with a thinner retina in the temporal perifoveal area in these patients [58]. Data comparison at the end of follow-up in our study cohort showed a significant trend to stabilization of the ophthalmologic parameters, mainly regarding the BCVA and OCT-derived measurements in the T2DM + DR + A/ω3 vs. the T2DM + DR − A/ω3 groups. This is an interesting point to consider because interventions directed to counteract several of the pathogenic mechanisms involved in the deleterious effects of chronic hyperglycemia on the retinal microvasculature are needed.

Moscos et al. [61] reported an increase in the foveal thickness of T2DM patients at two years of oral supplementation with carotenoids, suggesting that the above type of intervention may benefit the visual function in diabetics. It has also been said that early oral supplementation with nutraceuticals provides a significant health benefit to prevent DR progression because of its antioxidant and anti-inflammatory properties, reducing both the neural and vascular damage caused in the retina by the hyperglycemia [7,12,19,62,63]. The combined A/ω3 formula used herein strongly supported the descriptions of the above authors and our previous works [19,35,39,40,49] on the processes of retinal neuroprotection and vasculoprotection. Moreover, Amato et al. [64] emphasized the possible side effects of systemic neuroprotectants as well as doubts about the appropriate retinal bioavailability of these substances in contrast with the intravitreal injections. We hereby confirm that with the formulation used herein, no adverse effects were reported but scarcely mild gastrointestinal discomfort, while the intravitreal injections may potentially produce endophthalmitis, hemorrhages, rhegmatogenous retinal detachment, lens damage, and/or other complications [65]. These authors interestingly described the feasibility of delivering magnetic nanoparticles functionalized with a neuroprotectant substance by means of intravitreal injections in mice, suggesting an interesting role of these new approaches to protect the retina in diabetic patients. In this concern, Dolz-Marco et al. [66] also reported that intravitreal DHA administration (at the specific assayed doses) in rabbits is safe and potentially useful for treating retinal diseases.

No data from the interim time points were presented in this work because the main results from the 18-months follow-up were previously published [19]. Regarding the above data, the BCVA (each eye separately) was significantly higher in the CG as compared to the T2DM group [(CG at baseline: RE: 0.95 ± 0.10 and LE: 0.96 ± 0.08; *p* < 0.001/CG; at 18 months: RE: 0.95 ± 0.11 and LE: 0.95 ± 0.10; *p* < 0.001) (T2DMG at baseline: RE: 0.81 ± 0.30 and LE: 0.83 ± 0.22; *p* < 0.001/T2DMG; at 18 months: RE: 0.78 ± 0.22 and LE: 0.76 ± 0.22; *p* < 0.001)]. Regarding the redox status of the study population at 18 months, on the one hand, plasmatic pro-oxidants (MDA/TBARS levels) significantly decreased in the T2DM + A/ω3 subgroup with respect to the T2DM − A/ω3 patients, and on the other hand, the TAC significantly increased in the T2DM + A/ω3 subgroup, while the non-supplemented participants did not show any change. These data are similar to those obtained from the 38-month follow-up during the present work.

The authors are confident that the results obtained during this follow-up study can be useful in designing an integrative document with the clinical (ocular fundus multimodal imaging) and molecular-genetic biomarkers (MDA/TBARS, TAC, vit C, and *SLC23A2* gene) in the form of practical guidelines that can be helpful for primary care physicians as well as to ophthalmologists and ophthalmic researchers. In this concern, classical unmodifiable risk factors, such as the older age and/or longer DM duration have been widely involved in the development and progression of DR, altogether with the modifiable biochemical parameters of higher glycemia and HbA1c as well as the increased BMI and the lack of physical exercise [1,2,3,4,5,6,19]. All these, and the oxidative stress parameters reported herein (MDA/TBARS, TAC, vit C), can help outline the importance of integrating patient characteristics, as well as clinical and biochemical biomarkers better to identify diabetics at risk of retinopathy and visual disability. This follow-up study strongly emphasizes the benefits of the effects of the A/ω3 administration in mono-combination on both the DR and biochemical blood traits in T2DM patients. Up to today, our group continues to pursue research, consistently looking for new solutions to avoid diabetic blindness.

A study limitation is the relatively small final sample of participants distributed in each group and subgroup reaching the 38-month follow-up. Patients who developed PDR or DME were excluded from data processing to avoid the bias of statistical procedures, mainly because these patients needed to be immediately treated with specific medical, laser, or surgical interventions. Statistical processing of the GSH data showed surprising results. No significant differences were found at the end of the study regarding the A/ω3 supplementation (containing GSH). We may hypothesize that sample handling may induce artificial oxidation of GSH, producing GSSG that interfered with our results. It is needed to proceed with a rapid –SH alkylation with N-ethylmaleimide to improve these results for further research. The study has provided a large amount of data for easy analyses. Because of this, we have focused on the main objectives of our work, and some of the data have been omitted for the present study. In fact, the expression of the *SLC23A2* gene was only assayed at baseline in the main groups and subgroups of participants. No comparison was made of the *SLC23A2* gene expression between the baseline and the end of follow-up and nor in the participants assigned or not to the oral supplement regimen due to unsolvable problems at the end of follow-up. Nevertheless, the differential expression profile of the SLC23A2 gene in diabetics, as compared to the controls, as well as in diabetics with and without NPDR, provided interesting data at the beginning of the study (as reflected in Figure 4), showing outstanding correlation with the plasma vit C levels. In fact, Hierro et al. [67] demonstrated a SLC23A2 down-regulation in the kidney and brain from a model of streptozotocin Zucker rats.

## 5. Conclusions

In summary, the results of the present study reveal the importance of multicenter collaborative works in DR research to better managing the diabetic retina. As retinal microangiopathy is a sight-threatening complication of DM, early diagnosis, proper supervision, and appropriate treatment of DR are pivotal for optimizing eye and vision care in the affected patients. We confirm that OS plays relevant roles in DR initiation and progression, also suggesting that OS can be detected with easily accessible and inexpensive blood analyses of T2DM patients. We have identified the MDA/TBARS, TAC, vit C, and *SLC23A2* gene as presumptive prognostic biomarkers to improve knowledge on DR and ophthalmological outcomes. Large scale studies are needed to better estimate its efficiency and further usability in clinical practice. Finally, this 38-month follow-up study supports that a regular A/ω3 supplementation reduces the oxidative load and may serve as a dietary prophylaxis/adjunctive intervention for patients at risk of diabetic blindness

## Figures and Tables

**Table 1:** Inclusion and exclusion criteria for the study on diabetic retinopathy.

**INCLUSION**
Males/Females, aged > 25 and < 80 years with type 2 diabetes, as the TDM2 group.
Healthy individuals, as the CG.
No comorbidities. No ocular surgery or laser for 12 months (at least). No other oral supplements with antioxidants and/or omega 3 fatty acids, including vitamins in eyedrops.
Provided written informed consent before starting any related activities.
Participants able to attend the visits and to follow the study guidelines during the study period.
**EXCLUSION**
Males and females, aged < 25 years and > 80 years.
T1DM patients.
Patients with proliferative diabetic retinopathy, diabetic macular edema, or ocular or systemic diseases or aggressive treatments. Previous ocular surgery or laser for 12 months (at least). Other oral supplements with antioxidants and/or omega 3 fatty acids, including vitamins in eyedrops.
No acceptance for the study participation and/or not signing the informed consent. Unable to attend the visits or to follow the study guidelines during the study period.

DM, diabetes mellitus; TDM2, type 2 diabetes mellitus; T1DM, type 1 diabetes mellitus; CG: Control group.

**Table 2:** Demographics, lifestyle, and classical biochemical blood traits in the study participants. Similar age and distribution by gender, but significant differences regarding the glycemic profile were observed between the T2DM patient vs. the CG throughout the follow-up.

VARIABLES	T2DM		CG		*p*-Value
	*Baseline*	*38-Months*	*Baseline*	*38-Months*	*End of Study*
**Age** *Years*	60 (10)	65 (8)	55 (12)	60 (8)	0.765
**Gender** % *women*	51	54	47	58	0.841
**DM Fam. Hist.** %	60	62	37	35	0.00001 **
**DM duration** *Years*	14 (3)	18 (5)	-	-	-
**BMI** Kg/mm^2^	30 (3)	30 (4)	24 (3)	21 (3)	0.001 *
**Physical Ex.** %	38	35	42	43	0.916
**Glycemia** mg/dL	146 (62)	140 (8)	89 (12)	91 (3)	0.000001 **
**HbA1c** %	9 (1)	7 (1)	6 (0.3)	5 (0.3)	0.000001 **

T2DM: Type 2 diabetes mellitus; CG: Control group. DM: Diabetes Mellitus; BMI: Body mass index; HbA1c: Glycosilated Haemoglobin; Fam. Hist.: Family History; Physical Ex: Physical Exercise. Values are mean (SD) or %. *p*-value (* *p* < 0.05; ** *p* < 0.001) reflects statistical differences between the T2DM and CG of participants at the end of the study. Age, Glycemia, and HbA1c was compared using student *t*-test for unpaired samples. BMI was compared using the Mann–Whitney U test. Percentages of Gender, DM Fam. Hist., and Physical Ex. were compared using the Fisher exact test.

**Table 3:** Ophthalmologic data in the supplemented vs. the non-supplemented participants.

Variables	*T2DM*			*CG*			*p-Value*	
	Baseline	38-Months		Baseline	38-Months		End of Study	
		+A/ω3	−A/ω3		+A/ω3	−A/ω3	+A/ω3	−A/ω3
**BCVA RE** *decimal scale*	0.8 (0.1)	0.7 (0.1)	0.6 (0.1)	0.9 (0.1)	0.9 (0.1)	0.9 (0.2)	0.002 **	0.002 **
**BCVA LE** *decimal scale*	0.8 (0.2)	0.7 (0.1)	0.5 (0.1)	0.9 (0.1)	0.9 (0.2)	0.8 (0.1)	0.049 *	0.001 **
**IOP RE** mm Hg	15 (2)	15 (2)	15 (2)	15 (2)	14 (2)	15 (3)	0.415	0.432
**IOP LE** mm Hg	15 (2)	16 (2)	15 (2)	16 (2)	13 (2)	16 (2)	0.068	0.453
**CMT RE** µm	252 (32)	245 (29)	240 (28)	251 (33)	253 (35)	258 (40)	0.585	0.514
**CMT LE** µm	258 (55)	242 (37)	236 (46)	255 (36)	255 (43)	249 (35)	0.539	0.561

BCVA: Best-corrected visual acuity; RE: Right eye; LE: Left eye; IOP: Intraocular pressure; CMT: Central Macular Thickness; T2DM: Type 2 diabetes mellitus; CG: Control group. +A/ω3: Daily supplementation with antioxidants/omega 3 fatty acids; −A/ω3: No supplementation regimen. Values are mean (SD) for each eye. *p*-value (* *p* < 0.05; ** *p* ≤ 0.002) reflects statistical differences between the T2DM and CG of participants at the end of the study. Comparisons of BCVA and IOP were made using the Mann–Whitney U test. CMT was compared using the student t-test for unpaired samples.

**Table 4:** Diabetic Retinopathy course through the 38-months of follow-up.

T2DM Patients + DR (*n* = 129)						T2DM Patients − DR (*n* = 158)					
DR Impairment: 22%						DR Impairment: 27%					
Mild		Moderate		Severe		Mild		Moderate		Severe	
13%		5%		4%		12%		9%		5%	
+A/ω3	−A/ω3	+A/ω3	−A/ω3	+A/ω3	−A/ω3	+A/ω3	−A/ω3	+A/ω3	−A/ω3	+A/ω3	−A/ω3
3%	10%	2%	3%	1%	3%	3%	9%	2%	7%	2%	3%

T2DM: Type 2 diabetes mellitus; CG: Control group; +DR: With diabetic retinopathy; −DR: Without diabetic retinopathy; +A/ω3: Participants randomly assigned to the daily supplementation with antioxidants and omega 3 fatty acids; −A/ω3: No assigned to the supplementation regimen. Values are the percentages of DR impairment regarding the NDPR (non-diabetic retinopathy) clinical form. NDPR has been classified according to the ETDRS (early treatment diabetic retinopathy study) system [28] as a mild, moderate, or severe stage.

**Table 5:** Oxidative stress markers in the study participants.

VARIABLES	*T2DM 38-Months*				*CG 38-Months*		*p-Value*
	*+DR*		−*DR*		+A/ω3	−A/ω3	End of study
	+A/ω3	−A/ω3	+A/ω3	−A/ω3			
**MDA/TBARS** (µM)	3 (0.2)	3.7 (0.2)	2.7 (0.2)	3.0 (0.2)	1.6 (0.1)	2.0 (0.1)	0.001 **
**TAC** (mM)	1.8 (0.1)	1.1 (0.1)	2.0 (0.1)	1.5 (0.1)	3.2 (0.2)	2.8 (0.2)	0.001 **
**GSH** (µM)	1.4 (0.6)	0.9 (0.7)	1.6 (0.1)	1.4 (0.1)	2.7 (0.2)	2.3 (0.2)	0.842
**Vit C** (µmol/mL)	45 (18)	33 (15)	43 (16)	40 (21)	59 (20)	56 (20)	0.001 **

MDA/TBARS: Malondyaldehyde/Thiobarbituric acid reactive substances; TAC: Total antioxidant capacity; GSH: Total glutathione; Vit C: Vitamin C-Ascorbic acid; T2DM: Type 2 diabetes mellitus; CG: Control group. +A/ω3: Assigned to the daily supplementation with antioxidants and omega 3 fatty acids; −A/ω3: Not assigned to the supplementation regime. Values are mean (SD) for each eye. *p*-value (** *p* < 0.001) reflects statistical differences between the T2DM and CG of participants at the end of the study (38-month follow-up). All comparisons were made using the Mann–Whitney U test.

## Abbreviations

+/− A/ω3	plus/without a pill of nutraceutical supplement per day
+/− DR	with/without diabetic retinopathy
A	Antioxidants
BCVA	best corrected visual acuity
BMI	body mass index
CG	healthy control group
DHA	docosahexaenoic acid
DM	diabetes mellitus
DME	diabetic macular edema
EDTA	ethylene diamine tetra acetic acid
GSH	glutathione
GSSG	oxidized GSH
HbA1c	glycosylated haemoglobin
HPLC	high performance liquid chromatography
IOP	intraocular pressure
MedDiet	Mediterranean diet
MDA	malondialdehyde
NPDR	non proliferative diabetic retinopathy
OCT	optical coherence tomography
OS	oxidative stress
PDR	proliferative diabetic retinopathy
ROS	reactive oxygen species
SD-OCT	spectral domain optical coherence tomography
SLC23A2	solute carrier family 23 member 2
T1DM	type 1 diabetes mellitus
T2DM	type 2 diabetes mellitus
TAC	total antioxidant capacity
TBARS	thiobarbituric acid reactive substances
TNB	5-thionitrobenzoic acid
Vit	Vitamin
ω3	omega 3 fatty acids
